# Perceived Confidence in Obtaining an Alzheimer’s Disease Diagnosis: Exploring Race and Rurality

**DOI:** 10.1093/geroni/igaf122.4171

**Published:** 2025-12-31

**Authors:** Hannah Huff, Anita Reina, Caleb Snead, Lydia Burton, Don Scott, Stephen Correia, Lisa Renzi-Hammond, Jenay Beer

**Affiliations:** University of Georgia, Athens, Georgia, United States; Kennesaw State University, Kennesaw, Georgia, United States; Northwestern University, Evanston, Illinois, United States; University of Georgia, Athens, Georgia, United States; University of Georgia, Athens, Georgia, United States; University of Georgia, Athens, Georgia, United States; University of Georgia, Athens, Georgia, United States; University of Georgia, Athens, Georgia, United States

## Abstract

Alzheimer’s disease (AD) disproportionately affects African Americans, who are twice as likely as Caucasians to develop AD. Research shows; however, that despite higher likelihood of developing AD, African Americans are 35% less likely to get an AD diagnosis. Past research has identified structural barriers (e.g., medical underservice, out-of-pocket costs) that disproportionately affect African American communities, but the intersection of rurality and race on obtaining an AD diagnosis has not been well-studied. In this study, we investigate confidence in obtaining a dementia diagnosis among African American residents of both rural and urban communities. As part of a larger dementia needs assessment, a cross-sectional survey was administered to Georgians (N = 531), assessing confidence in getting a dementia-related diagnosis if they started having memory concerns. Surveys were administered at health fairs and community-focused education/programming. Self-reported confidence in obtaining a dementia diagnosis for rural and urban communities combined was greater for African Americans (mdn=4.00; n = 131) compared to Caucasians (mdn=3.00; n = 400), U = 31420.50, p < 0.001, r = 0.15. Among rural-dwelling participants, confidence in obtaining a dementia diagnosis was greater for African Americans (mdn=4.00; n = 101) compared to Caucasians (mdn=3.00; n = 267), U = 16949.50, p < 0.001, r = 0.20. Additionally, rural African Americans (mdn=4.00, n = 101) expressed greater confidence than urban African Americans (mdn=3.00, n = 30), U = 1068.00, p = 0.011, r = 0.22. Although this study indicates rural-dwelling African Americans are confident in obtaining an AD diagnosis, previous studies suggest they lack trust in healthcare providers. Future research on the intersection of race and rurality should attempt to understand why rural African Americans are more confident than Caucasian counterparts.

